# Antibacterial Activity of Marine and Black Band Disease Cyanobacteria against Coral-Associated Bacteria

**DOI:** 10.3390/md9102089

**Published:** 2011-10-24

**Authors:** Miroslav Gantar, Longin T. Kaczmarsky, Dina Stanić, Aaron W. Miller, Laurie L. Richardson

**Affiliations:** 1 Department of Biological Sciences, Florida International University, Miami, FL 33199, USA; E-Mails: gantarm@fiu.edu (M.G.); aaron.miller@fiu.edu (A.W.M.); 2 St. John’s River State College, St. Augustine, FL 32084, USA; E-Mail: Longin.Kaczmarsky@fiu.edu; 3 Institute Department of Aquatic Microbiology, Institute for Biodiversity and Ecosystem Dynamics, University of Amsterdam, Amsterdam1098 XH, The Netherlands; E-Mail: D.Stanic@uva.nl

**Keywords:** antimicrobial activity, cyanobacteria, coral disease

## Abstract

Black band disease (BBD) of corals is a cyanobacteria-dominated polymicrobial disease that contains diverse populations of heterotrophic bacteria. It is one of the most destructive of coral diseases and is found globally on tropical and sub-tropical reefs. We assessed ten strains of BBD cyanobacteria, and ten strains of cyanobacteria isolated from other marine sources, for their antibacterial effect on growth of heterotrophic bacteria isolated from BBD, from the surface mucopolysaccharide layer (SML) of healthy corals, and three known bacterial coral pathogens. Assays were conducted using two methods: co-cultivation of cyanobacterial and bacterial isolates, and exposure of test bacteria to (hydrophilic and lipophilic) cyanobacterial cell extracts. During co-cultivation, 15 of the 20 cyanobacterial strains tested had antibacterial activity against at least one of the test bacterial strains. Inhibition was significantly higher for BBD cyanobacteria when compared to other marine cyanobacteria. Lipophilic extracts were more active than co-cultivation (extracts of 18 of the 20 strains were active) while hydrophilic extracts had very limited activity. In some cases co-cultivation resulted in stimulation of BBD and SML bacterial growth. Our results suggest that BBD cyanobacteria are involved in structuring the complex polymicrobial BBD microbial community by production of antimicrobial compounds.

## 1. Introduction

Black band disease (BBD) of corals is known to contribute to the degradation of coral reefs in the wider Caribbean [1], the Indo-Pacific [2], including the Great Barrier Reef [3], and the Red Sea [4]. It has been reported to affect 70 species of corals, including both scleractinians and gorgonians [2], and can cause whole colony mortality by rapid tissue lysis. The disease often targets the ecologically important reef-framework coral species.

In appearance, BBD is a dark, well-defined cyanobacterial mat that forms a band, which moves across the coral surface, degrading coral tissue and leaving behind bare coral skeleton. It is a polymicrobial disease dominated by non-heterocystous filamentous cyanobacteria and contains populations of sulfate-reducing bacteria, sulfide-oxidizing bacteria, and heterotrophic bacteria. The BBD microbial community is highly diverse as revealed through microscopy [5,6] and, in particular, molecular biological methods [7–10].

It has been proposed that some of the BBD microbial diversity may be due to incorporation of coral surface mucopolysaccharide layer (SML) bacteria into the BBD population [8]. In turn, the presence of disease may affect the SML bacterial community. Previous studies of coral-associated bacteria revealed that apparently healthy corals contain less diverse bacterial communities than the healthy areas of diseased corals, and that the compositions of the two populations are different [7]. While it is known that coral-associated bacteria are present in coral tissue and skeleton as well as the SML, studies of the composition of coral-associated bacterial communities have focused on those present in coral tissue [7,8,11–15], with fewer studies targeting those within the coral SML [10,15,16]. The results of these studies, in general, indicate that members of the gamma- and alpha-proteobacteria dominate coral-associated bacterial populations.

The coral probiotic hypothesis [11] proposes that coral resistance to disease can be promoted by coral-associated bacteria, which prevent colonization by potential pathogens or outcompete pathogens which may settle on coral. The protective mechanism of coral probiotic bacteria is proposed to include antibacterial activity, and to date there have been a number of studies aimed at demonstrating that corals and their associated bacteria possess such properties [16–22]. Koh [18] showed that the alcohol extract of a large percentage of coral samples had antibacterial activity against a number of heterotrophic bacteria and cyanobacteria, while results obtained by Kim [20] demonstrated antibacterial activity for both polar and non-polar (coral-derived) fractions with higher activity associated with non-polar fractions. In both studies the extracts were prepared from the coral holobiont (the coral animal, endosymbiotic zooxanthellae, and coral-associated bacteria), and the origin of the active compounds was not determined. Ritchie [16] found antibacterial activity among the microbial community associated with the mucus of healthy corals.

Deciphering the relationships among and between the bacterial members of the complex BBD microbial consortium could provide insight into the etiology of this polymicrobial disease. Since cyanobacteria are the dominant component of BBD in terms of biomass, we selected this group of microorganisms to investigate their ability to inhibit growth of both BBD and (healthy) coral SML bacterial isolates. In this way, we hypothesize that cyanobacteria may structure the complex BBD microbial community. We also investigate, for comparison, the antibacterial activity of sub-tropical marine cyanobacteria from other marine sources. This study assesses the potential of marine cyanobacteria as a source for novel antibacterial agents that could potentially be applied in human health.

## 2. Results

### 2.1. Inhibition of BBD and SML Bacteria during Co-Cultivation with Cyanobacteria

**Figure 1:**
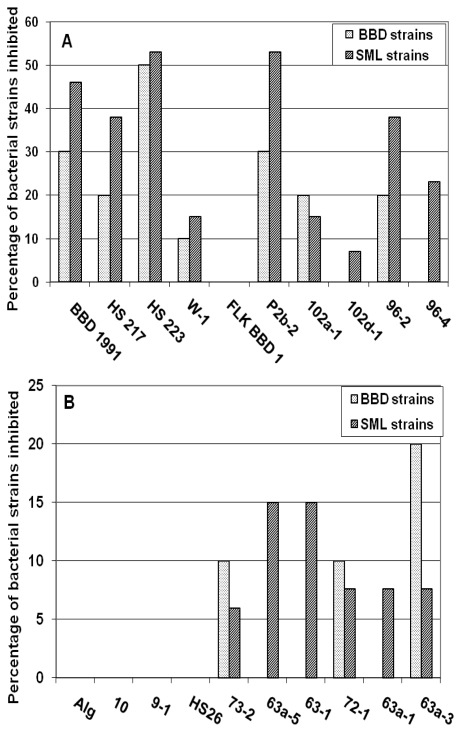
Percent of black band disease (BBD) and surface mucopolysaccharide layer (SML) bacteria inhibited by individual cyanobacterial strains in co-cultivation experiments. (**A**) BBD cyanobacteria; (**B**) other marine cyanobacteria. Note different scales on Y axes.

Co-cultivation of BBD and SML bacteria with cyanobacteria revealed that, of the 20 cyanobacterial strains tested, 15 had antibacterial activity against at least one of the target bacteria. Nine of the BBD cyanobacterial strains, and six of the other marine cyanobacterial strains, exhibited activity (Figure 1).

**Figure 2:**
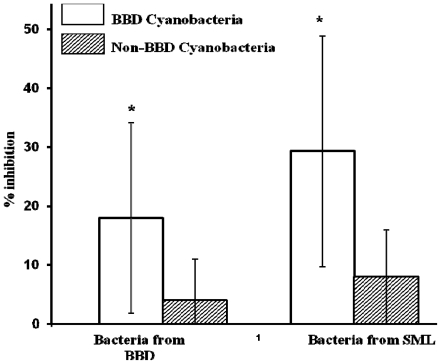
Inhibition of BBD and SML bacteria by BBD and other marine cyanobacteria in the co-cultivation experiment. Cyanobacteria from BBD inhibited significantly more bacteria from both BBD (*t*-test: *P* = 0.026) and SML (Mann-Whitney test: *P* = 0.016) than other marine cyanobacteria. Error bars = SD (non-transformed data). Asterisks indicate statistically significant results.

Seven BBD cyanobacteria were active against both BBD and SML bacteria, while only three of the other marine cyanobacteria were active against both groups. Each of the active cyanobacterial strains from both sources was active against SML bacteria. In contrast, only seven of the nine active BBD and three of the six active other marine cyanobacterial strains were active against BBD bacteria. Overall, co-cultivation with BBD cyanobacteria resulted in a higher, statistically significant number of inhibitions than other marine cyanobacteria (Figure 2).

### 2.2. Interactions between Individual Bacterial and Cyanobacterial Strains

The activity of individual cyanobacterial strains against individual bacterial strains is summarized in Table 1. This data set includes, in addition to antibacterial activity, cases in which stimulation of bacterial growth was observed (strain designations bolded). The results of experiments examining both co-cultivation and exposure to lipophilic extract are summarized. Strain abbreviations designated in Table 2 as B (BBD), S (SML) and P (known pathogen) were used in Table 1 for ease of comparison of results for these three categories of test bacteria.

Of the 460 combinations tested (20 cyanobacterial strains × 23 bacterial strains), co-cultivation with cyanobacteria resulted in inhibition in 11% of cyanobacteria/BBD bacteria tests and 18% of cyanobacteria/SML bacteria tests, with stimulation observed for 1% and 4% respectively (Table 1). In many cases the same bacterial strain was inhibited by different cyanobacteria. Five of the bacterial isolates tested (four SML and one BBD) exhibited very high sensitivity to co-cultivation with BBD cyanobacteria. For example strain B6 (isolate HS-217-2g, identified in GenBank as *Alteromonas* sp.) was inhibited by seven of the 10 BBD cyanobacteria. In contrast, none of the other marine cyanobacteria affected growth of this isolate. Of the SML bacterial isolates, strains S3, S6, S7, and S13 (three *Vibrio* sp. and one *Bacillus*—see Table 2) were each inhibited during co-cultivation with five to seven BBD cyanobacteria (Table 1).

### 2.3. Activity of Cyanobacterial Extracts

**Figure 3:**
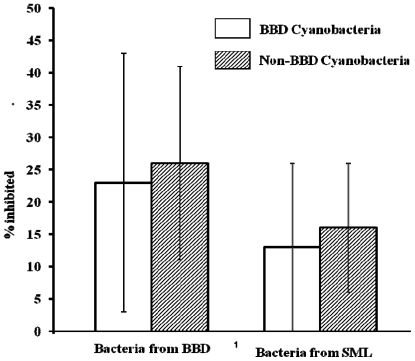
Inhibition of BBD and SML bacteria by lipophilic extracts from BBD and other marine cyanobacteria. There were no significant differences between these two groups of cyanobacteria. Error bars = SD (non-transformed data).

Exposure of test BBD and SML bacteria to lipophilic cyanobacterial extracts (Table 1) resulted in overall higher inhibition when compared to co-cultivation. There were no cases of growth stimulation. Lipophilic extracts of nine of the 10 of both BBD and other marine cyanobacteria were active (18 of the 20 strains). Overall, 23% of BBD bacteria and 26% of SML bacteria exhibited inhibition of growth after exposure to lipophilic extracts. There was no significant difference in the activity of BBD and other marine cyanobacteria lipophilic extracts against the BBD and SML bacteria tested (Figure 3).

The lipophilic extracts exhibited much more antimicrobial activity than the hydrophilic extracts, which in most cases did not have any activity (data not shown). Hydrophilic extracts of only three of the nine BBD cyanobacteria tested were active, with two strains inhibiting one of the 10 BBD strains (B1 and B7) and the third inhibiting one SML strain (S8). Hydrophilic extracts of other marine cyanobacteria did not inhibit any BBD strains, although four of these other marine cyanobacteria (seven of 10 tested) did inhibit SML bacteria. Of these, three cyanobacteria inhibited one strain and one cyanobacterium inhibited two strains, with three strains active against S12.

### 2.4. Activity of Extracts *vs.* Co-Cultivation with SML and BBD Bacteria

In Table 1, it can be seen that members of all cyanobacterial genera tested exhibited antibacterial activity, either during co-cultivation or exposure to lipophyllic extracts. The antibacterial activities of cyanobacterial extracts did not coincide with the results of co-cultivation experiments using the same cyanobacterial strains. The most dramatic example is the case of the non-BBD *Leptolyngbya* isolates, in which none of the co-cultivation experiments yielded any effect whereas lipophilic extracts inhibited nine strains of BBD bacteria and seven of SML bacteria.

### 2.5. Antibacterial Activity against Known Coral Pathogens

Only one of the three known coral pathogens tested, *Vibrio shiloi* (P3 in Table 1), was inhibited by co-cultivation with cyanobacteria, and only one of the 20 cyanobacterial strains tested (BBD *Geitlerinema* HS 223) elicited this result. None of the cyanobacterial extracts elicited growth inhibition of *Vibrio shiloi*, and no assay (co-cultivation or extract) elicited stimulation of this pathogen. The other two known coral pathogens tested, *Aurantimonas coralicida* and *Serratia marcescens*, were affected (inhibition only) by lipophilic cyanobacterial extracts (Table 1), with no effect observed for the hydrophilic extracts tested (not shown). Lipophilic extracts of four of the BBD cyanobacterial strains and three other marine cyanobacterial strains inhibited *A. coralicida*, while lipophilic extracts of three BBD cyanobacteria and six other marine cyanobacteria inhibited *S. marcescens* (Table 1). Overall there were 16 cases of inhibition of both pathogens, with five of the cyanobacterial strains (including both BBD and other marine cyanobacteria) inhibiting both *A. coralicida* and *S. marcescens* (P1 and P2 in Table 1).

## 3. Discussion

In recent years the study of coral-associated microorganisms has greatly expanded, with a focus on the role of such microorganisms in coral health and disease. A number of these studies have documented antibacterial activity among and between coral-associated bacteria [16,21], and also antimicrobial activity of corals themselves [16,22].

Cyanobacteria are well known to produce antibacterial compounds [23–26] and are known to be associated with corals [8]. However, little work has been done to assess the antibacterial activity of coral-associated cyanobacteria, or, for that matter, cyanobacteria in other marine environments. In this work we assessed the activity of marine cyanobacteria on growth of coral-associated bacteria using two different methods: co-cultivation in which cyanobacterial metabolites were allowed to diffuse into agar (without breaking their cell walls), and use of extracts prepared from equal amounts of dried cyanobacterial culture biomass. Our results revealed that co-cultivation of cyanobacteria with BBD or SML heterotrophic bacteria resulted in 4.8% cases of inhibition of BBD bacteria and 10% cases of inhibition of SML bacteria. This result was statistically significant for both BBD and other marine cyanobacteria (*P* < 0.05; see legend of Figure 2).

Overall, BBD bacteria appear to be more resistant to antibacterial activity of marine cyanobacteria in general. This might be expected since BBD bacteria live in close physical contact with cyanobacteria. Within the total BBD bacterial/cyanobacterial test cases, 82% of BBD bacteria exhibited resistance. However, the 18% that were inhibited were represented by six of the 10 BBD bacterial strains tested. This result may be important in that the complex BBD microbial community may require small populations of some functional members; from this perspective, regulation of growth of certain bacterial members within the organic carbon/nutrient rich BBD environment would be an important part of the disease etiology.

There was a much higher percentage (30% of test cases) of inhibition of SML bacteria by BBD cyanobacteria when compared to inhibition of BBD bacteria. This finding suggests that BBD cyanobacteria may be capable of eliminating potentially beneficial, protective coral-associated bacteria, thus supporting the coral probiotic hypothesis [11]. Alternatively, BBD cyanobacteria may eliminate SML bacteria within a developing BBD microbial community to reduce competition. Other marine cyanobacteria also showed antibacterial activity in 9% of test cases. Therefore, it can be hypothesized that non-pathogenic cyanobacteria present in healthy corals may also provide protection against disease-causing microorganisms.

We observed, to a lesser extent, stimulation of growth of some bacterial isolates during co-cultivation with cyanobacteria, in particular with other marine cyanobacteria. While there were only two cases of stimulation by BBD cyanobacteria (both of SML bacteria), there were 10 cases of stimulation by other marine cyanobacteria. This is in agreement with the findings of Morrow *et al.* [27] who reported that two marine *Lyngbya* species stimulated, but did not inhibit, the growth of marine bacteria.

Antimicrobial activity was found mostly among extracts obtained with non-polar rather than hydrophilic solvents, which is in agreement with the findings of others [28]. This can be attributed to the fact that lipophilic compounds more easily cross the cell membrane, thus are more likely to exert an affect. BBD cyanobacteria appear to produce a variety of antibacterial compounds that are not excreted, thus their effect on members of the BBD consortium would be limited. We believe that the co-cultivation experiments more closely mimic the ecological conditions within BBD since microscopic observations of BBD samples reveal healthy, highly motile cyanobacteria and an absence of lysing filaments.

Comparison of the coral-associated bacterial isolates used in these experiments with a recently conducted meta-analysis of 84 BBD bacterial clone libraries [29] revealed that none of our isolates was a match to BBD sequences deposited from these sources in GenBank. This is not unusual, as pointed out recently in a study of coral-associated bacteria by Rypien *et al.* [21] that used a combination of culture-based and molecular methods. We do note that our test bacteria were represented by members of three genera, *Marinobacter* sp., *Alteromonas* sp., and *Vibrio* sp., which have been reported in BBD clone libraries [29]. Of these, the BBD *Marinobacter* isolate used in the current study (strain B5) and the *Alteromonas* isolate (strain B6) were inhibited by two and seven strains of BBD cyanobacteria respectively (Table 1). Neither was inhibited by other marine cyanobacteria, and neither was stimulated by any (BBD or other marine) cyanobacterial strain tested. The *Vibrio* sp. strains were inhibited to different extents by both BBD and other marine cyanobacteria.

For the known coral pathogens, co-cultivation in only one of the 60 tests (20 cyanobacteria × 3 bacterial pathogens) resulted in inhibition. This result was obtained for the bacterial bleaching pathogen *Vibrio shiloi*. Nissimov *et al.* [30] found, in an investigation of the antibacterial properties of bacteria from coral mucus, that 5.7% of bacterial isolates inhibited *Vibrio shiloi*. Therefore, if this bacterium is responsible for bleaching of corals under certain environmental conditions, as proposed by Rosenberg *et al.* [31], it appears that protection of coral would be not be conferred by cyanobacterial members of the coral holobiont, and perhaps not by bacterial members.

It was surprising that while co-cultivation did not inhibit growth of the other two coral pathogens tested (*Aurantimonas coralicida* and *Serratia marcescens*) there was inhibition in 27% (16 of 60) of the tests conducted using lipophilic cyanobacterial extracts. Therefore cyanobacteria (both BBD and other marine) may potentially be involved in protecting corals from these specific pathogens.

Our results indicate that both pathogenic (BBD) and non-pathogenic marine cyanobacteria can affect the growth of coral-associated bacteria, and that while most of this activity is manifested as inhibition of growth, growth stimulation also occurs. While we did not, in the present study, identify the cyanobacterial compounds responsible for the observed activity, other studies in our laboratory suggest that at least some of the activity could be due to the cyanotoxin microcystin. We have previously shown that BBD cyanobacteria produce microcystins [32–34] and that freshly collected BBD samples contain microcystins [32]. All of the cyanobacteria used in the current study were previously shown to produce microcystin-LR in the laboratory [33,34], with the exception of two *Leptolyngbya* strains that were not tested (strains 96-2 and 9-1; see Table 3). We have also shown that exposure of healthy coral fragments to low concentrations (1 μg L^−1^) of purified microcystin-LR resulted in increased bacterial growth in coral fragments, observed using Scanning Electron Microscopy [35]. This observation was investigated by exposing bacterial isolates to three concentrations (1, 100 and 500 μg L^−1^) of purified microcystin-LR and monitoring growth. These experiments were carried out using 24 of the 26 bacteria used in the present study (the two strains not tested were SML strains 1-1 and 1-14, both *Vibrio* spp.). Results were variable, with microcystin exposure both inhibiting and stimulating bacterial growth. When comparing the microcystin results with the results of the current study, only 17% of the cases of inhibition or stimulation by co-cultivation, and 23% of the cases of inhibition or stimulation in response to exposure to lipophilic extracts, were recorded for strains that responded to microcystin. Separating microcystin effects from other compounds would require detailed chemical analyses. Another potential source of the active compounds documented in this study consists of bacteria associated with the non-axenic cyanobacterial cultures. However, we deem this source to be negligible since all cyanobacteria were grown autotrophically for these experiments, thus their biomass would be orders of magnitude higher than the contaminants. Microscopic examination of the cyanobacterial cultures did not reveal the presence of bacteria; they could be detected only by plating cyanobacteria onto heterotrophic media.

## 4. Experimental Section

### 4.1. Source of Cyanobacterial and Bacterial Isolates

Table 3 presents the source and identities of the 20 cyanobacteria investigated in this study. Ten cyanobacterial strains from BBD, representing members of three cyanobacterial genera, from reefs of the Florida Keys, Bahamas, and the Philippines, and ten other marine cyanobacterial strains representing four genera from the Gulf of Mexico and reefs of the Florida Keys, were obtained as non-axenic uni-algal cultures as previously described [33,37]. The three BBD cyanobacterial genera have been detected in multiple BBD mats on different reefs of the Caribbean and the Philipines and from different coral host species [37]. From work by our group and others it appears that specific cyanobacteria are found in BBD regardless of host or location [7,9,37,39,40] hence our selection of cyanobacterial isolates from the different sources. The other marine cyanobacteria included mat-forming, filamentous types and planktonic, unicellular types from sub-tropical reef habitats in Florida as well as the Gulf of Mexico. All filamentous cyanobacteria were cultured from individual trichomes and were uni-algal cultures. Accompanying bacteria were closely associated with the cyanobacterial sheaths. Cultures of unicellular cyanobacterial isolates were axenic.

Heterotrophic bacteria used as test strains were isolated from BBD infections on the host coral *Siderastrea siderea* on Horseshoe Reef, Lee Stocking Island, Bahamas (10 strains), and from the SML of apparently healthy colonies of *S. siderea* on reefs in the Florida Keys (13 strains), summarized in Table 2. Unlike BBD cyanobacteria, for which the same 16S rRNA gene sequences are found on different coral host species and in different geographical regions [37], BBD-associated heterotrophic bacteria have been shown to be region specific [40]. On the other hand, Caribbean coral-associated heterotrophic bacteria have been reported to be coral host species specific [13]. Therefore we selected test SML bacteria from different colonies of the same coral species (*S. siderea*) to decrease variability based on host specificity. Additionally, three known bacterial coral pathogens were tested. These were *Aurantimonas coralicida*, *Serratia marcescens*, and *Vibrio shiloi*, associated with the coral diseases white plague type II, white pox, and bacterial bleaching [41–43] respectively, and were available as previously isolated laboratory cultures.

### 4.2. Field Sampling

Samples of BBD and the SML of apparently healthy corals were collected, using sterile needleless 10 mL syringes, while SCUBA diving. After collection, samples were maintained at ambient seawater temperature in darkness until return to the lab. Strains of heterotrophic bacteria were isolated into pure culture by streaking BBD or SML samples onto Marine Agar (Difco) plates. Inoculated plates were incubated at room temperature and colonies with different morphologies were picked and replated to purity. Pure cultures were maintained at room temperature on marine agar slants, while cyanobacterial cultures were maintained on marine BG11 liquid medium in 125 mL Erlenmeyer flasks at 26 °C, under a 12:12 h light:dark fluorescent light regime with an intensity of 20 μE m^−2^ s^−1^.

Taxonomic identifications were based on 16S rRNA gene sequencing and BLASTN search for the closest relatives in GenBank. Isolation of total genomic DNA, 16S rRNA gene amplification, and sequencing were performed as described elsewhere [37,44]. Cyanobacterial strains 96-2, Alg, and 9-1 were not sequenced and their taxonomic identification was based on morphology [45]. The sequence obtained from strain HS26 was most closely related (97% similarity) to an “uncultured cyanobacterium, clone Ct-3-39” obtained from coral reef sediments (accession number AM177427 in the GenBank database). This strain was also identified using classical methods based on morphology [45].

### 4.3. Co-Cultivation of Cyanobacteria and Heterotrophic Bacteria

One of the two methods used to assess the effect of the cyanobacterial isolates on growth of heterotrophic bacteria was the co-cultivation method [46]. BG11 agar plates were first overgrown with a test strain of cyanobacteria, then cut into 7 mm diameter disks which were transferred to empty Petri plates. Plates were then overlaid with warm Marine Agar and refrigerated overnight to allow diffusion of metabolites from the disk into the surrounding medium. Subsequently, each plate was spread-inoculated with one of the test bacteria, each of which had been grown in Marine Broth for 24 h prior to inoculation. This procedure was carried out for each combination of cyanobacteria-heterotrophic bacteria strains (20 × 26 = 520) and was carried out in triplicate (1560 individual tests). Plates were incubated at 27 °C for 48 h after which they were checked for the presence of inhibition zones or enhanced growth (stimulation). Antibacterial activity was recorded as positive if the zone of inhibition was equal to or greater than 9 mm in diameter.

### 4.4. Preparation of Cyanobacterial Extracts

The second method used to assess cyanobacterial/bacterial interactions was based on testing of cyanobacterial cell extracts. To prepare extracts cyanobacteria were grown in 3-L cultures in marine BG11 medium [47] at 28 °C and a light intensity of 30 μE m^−2^ s^−1^. Cultures were aerated with sterile air and harvested after four weeks by centrifugation. The collected biomass was freeze dried and kept at −20 °C until used for extract preparation as described in [46]. Two types of crude extracts were prepared, lipophilic and hydrophilic, from each sample. The biomass (100 mg of freeze dried sample for each cyanobacterial culture) was first extracted with chloroform (lipophilic extract), and then with 30% ethanol (hydrophilic extract). Both extracts were evaporated to dryness, then resuspended in absolute ethanol and 30% ethanol, respectively, to produce a final concentration of 1 mg of dry residue per mL of solvent.

### 4.5. Activity of Cyanobacterial Extracts

The agar diffusion technique was used to test the effect of cyanobacterial extracts on the growth of bacterial isolates. Wells in Marine Agar plates were made using a sterile glass tube (7 mm diameter) and filled with 70 μL of extract, with each extract tested in triplicate. Plates were dried in a laminar flow cabinet. Control plates contained solvent only (100% or 30% ethanol) and had no effect on the growth of any of the test bacteria. Once the plates were dry (no solvent present in the wells) they were spread inoculated with 24 h cultures of each of the test bacteria (grown in Marine Broth) and incubated at 27 °C for 48 h, after which the presence or absence of zones of inhibition, or stimulation of growth around wells, were recorded. Using this method it was apparent that there was very little activity of the hydrophilic extracts. Therefore, while the complete set (20 cyanobacteria × 26 bacteria in triplicate = 1560 assays) of cyanobacteria/bacteria combinations was tested for the lipophilic extracts, only nine of the 10 BBD cyanobacteria and seven of the 10 other marine cyanobacteria, were tested (1104 assays). All three known pathogens were also tested. All tests were performed in triplicate.

### 4.6. Statistical Analysis

To test for statistical significance of results, the percentages of inhibition of bacterial test strains by each cyanobacterial strain were first transformed to arcsin values. *T*-tests were used when the data passed both a Kolmogorov-Smirnov test for normality and a test for equal variance. If they failed a Mann-Whitney Rank Sum test was used. Statistical software used was SigmaStat ver. 3.5 (Systat Software, Inc.).

## 5. Conclusions

Marine environments, including tropical and sub-tropical coral reefs, have served as a source for novel natural products for decades. It may be that corals, and in particular their associated microorganisms, are a potential new source for antibacterials and other metabolites that could serve as biomedical agents with relevance to human health in a manner similar to bioactive compounds derived from marine sponges. In this study we have shown that cyanobacteria associated with a coral disease, and also non-pathogenic cyanobacteria from different sub-tropical marine environments, produce bioactive compounds that suppress the growth of coral-associated bacteria. These results supplement our earlier work that has shown that coral reef cyanobacteria produce the cyanotoxin microcystin. In terms of the microbial ecology of coral disease, specifically the subject of our study, black band disease, it appears that the cyanobacteria that dominate BBD lesions have an active roll in disease etiology based on production of the cyanotoxin microcystin, which can cause coral tissue lysis [35], and antibacterial agents that target bacteria both within the complex BBD polymicrobial disease consortium and within the protective SML of healthy coral colonies. Thus cyanobacterial toxin and antibacterial production appear to be integral parts of the pathobiology of this coral disease.

## Figures and Tables

**Table 1:** Effect (antibacterial activity and stimulation of growth) of BBD and other marine cyanobacteria on coral-associated bacteria. Stimulation is indicated in bold. Strain designations are defined in Table 2.

Cyanobacteria		Bacterial strains inhibited or stimulated by co-cultivation	Bacterial strains inhibited by lipophilic extracts
**BBD Isolates**			

BBD 1991	*Geitlerinema*	B3, B5, B6, S1, S3, S5, S7, S9, S11	B1, B2, B5, B9, B10, P2, P2, S2, S8, S12
HS 217	*Geitlerinema*	B3, B6, S5, S6, S7, S8, S13	P1
HS 223	*Geitlerinema*	B1, B3, B5, B6, B8, P3, S1, S3, S4, S6, S7, S9, S13	B1, B2, B3, B5, B9, B10, P1, P2, S2, S3
W-1	*Geitlerinema*	B6, S6, S7	B9, B10, S9, S10
FLK BBD1	*Leptolyngbya*	No effect	B1, B2, B5, B9, P1, P2, S3, S6, S8
P2b-2	*Leptolyngbya*	B1, B3, B6, S3, S4, S5, S6, S7, S11, S13	B5, B9, S1, S4, S7, S9, S10
102a-1	*Leptolyngbya*	B1, B6, S3, S7	B4
102d-1	*Leptolyngbya*	**S2**, **S9**, S13	B4
96-2	*Leptolyngbya*	B2, B6, S3, S6, S7, S8, S13	No effect
96-4	*Spirulina*	S3, S4, S9	B4, S2, S10

**Other marine isolates**			

Alg	*Leptolyngbya*	No effect	B2, B3, B4, B5, B9, P1, P2, S2, S4, S8, S12
10	*Leptolyngbya*	No effect	B9, P2, S4
9-1	*Leptolyngbya*	No effect	B5, B9, P1, S12
HS 26	*Leptolyngbya*	No effect	B5, S12
73-2	*Phormidium*	B1, **S2**, S8, **S9**	B1, B4, B5, P2, S1, S2, S4
63a-5	*Pseudanabaena*	S6, S7	B5, B10, P2, S4, S9, S10
63-1	*Pseudanabaena*	S4, S5, **S9**	No effect
72-1	*Pseudanabaena*	B3, S4	B5, B9, B10, P2, S7, S9, S10
63a-1	*Synechococcus*	**B1**, **B2**, **S2**, S4, **S5**, **S9**, **S10**	B1, B2, B3, B5, B9, P1, P2, S2, S9
63a-3	*Synechococcus*	B1, B2, S5, **S9**	B5, B9, B10, S7, S9, S12

**Table 2:** Bacterial strains used as target organisms for assessment of the effects of growth in co-culture with, or exposure to extracts of, BBD and other marine cyanobacteria.

Strain designation	GenBank closest relative	Accession No. of closest relative 1	Strain abbreviation 2
**Strains from BBD**			

HS-216-1a	*Vibrio harveyi*	AY750576	B1
HS-216-3d	*Bacillus megaterium*	AJ17381	B2
HS-217-1a	*Bacillus cereus*	AY305275	B3
HS-217-1c	*Photobacterium eurosenbergii*	AJ842344	B4
HS-216-4f	*Marinobacter* sp.	AY196982	B5
HS-217-2g	*Alteromonas* sp.	AY626838	B6
HS-216-4g	*Marinobacter aquaeolei*	AJ000726	B7
HS-216-4i	*Idiomarina* sp.	AB167047	B8
HS-217-2d	*Vibrio harveyi*	AY750575	B9
HS-216-4a	*Methylarcula* sp.	AJ534208	B10

**Known coral pathogens**			

	*Aurantimonas coralicida*	N/A	P1
	*Serratia marcescens*	N/A	P2
	*Vibrio shiloi*	N/A	P3

**Strains from healthy corals**			

1-1	*Vibrio* sp.	EU267634	S1
1-2	*Alcanivorax* sp.	EU781516	S2
1-3	*Vibrionaceae*	EF584057	S3
1-7	*Vibrio* sp.	EU267643	S4
1-8a	*Bacillus* sp.	EU070391	S5
1.8b	*Bacillus* sp.	FJ461465	S6
1-9	*Vibrio harveyi*	DQ995240	S7
1-10	*Vibrio* sp.	EU267643	S8
1-11	*Vibrio* sp.	FJ178079	S9
1-12	*Vibrio* sp.	EF100710	S10
1-13	*Vibrio* sp.	FJ457416	S11
1-14	*Vibrio* sp.	EU276991	S12
1-16	*Vibrio* sp.	EF584084	S13

1 Strains were 99% similar to the GenBank Accession No. listed with the exception of strains 1-1, 1-7 and 1-10 (100%) and HS-216-4a (96%);

2 Strain abbreviations used in Table 1. Abbreviations indicate source: B = BBD, S = SML, and P = known pathogen. N/A = not applicable as known cultures of these strains were used.

**Table 3:** Cyanobacterial isolates investigated, taxonomic identification, location of collection, and source of origin.

Isolate	Closest relative	Similarity %	GenBank access. No.	Location/Source	Reference
**BBD Isolates**					

BBD 1991	*Geitlerinema*^1^	99	DQ151461	Florida Keys, Algae Reef, BBD on *Montastraea annularis*	[36]
HS 217	*Geitlerinema*	99	EF110974	LSI Bahamas, Horseshoe Reef, BBD on *Siderastrea siderea*	[37]
HS 223	*Geitlerinema*	99	DQ680351	LSI Bahamas, Horseshoe Reef, BBD on *Siderastrea siderea*	[37]
W-1	*Geitlerinema*	99	EF154084	Florida Keys, Watson’s Reef, BBD on *Siderastrea siderea*	[37]
FLK BBD1	*Leptolyngbya*	98	EF110975	Florida Keys, South Carysfort, BBD on *Montastraea annularis*	[37]
P2b-2	*Leptolyngbya*	98	EF372581	Philippines, BBD on *Porites lutea*	[37]
102a-1	*Leptolyngbya*	97	EU743966	Florida Keys, BBD on *Dendrogyra cylindrus*	[33]
102d-1	*Leptolyngbya*^2^	97	EU743968	Florida Keys, BBD on *Montastraea annularis*	[33]
96-2	*Leptolyngbya*^3^,^4^	N/A	N/A	Florida Keys, BBD on *Montastraea annularis*	This work
96-4	*Spirulina*	93	EU743969	Florida Keys, BBD on *Montastraea annularis*	[33]

**Other marine isolates**					

Alg	*Leptolyngbya*^3^,^4^	N/A	N/A	Florida Keys, Algae Reef, mat on sediment	[33]
10	*Leptolyngbya*	99	FJ232377	Florida Keys, NN Dry Rocks, mat on *Montastraea cavernosa*	[33]
9-1	*Leptolyngbya*^3^,^4^	N/A	N/A	Florida Keys, Horseshoe reef, mat on *Colpophyllia natans*	This work
HS26	*Leptolyngbya*	N/A	FJ232376	Florida Keys, Horseshoe reef, mat on coral away from BBD	[33]
73-2	*Phormidium*	97	EU196366	Gulf of Mexico, plankton	[38]
63a-5	*Pseudanabaena*	97	FJ026734	Florida Keys, mat on sediment	[33]
63-1	*Pseudanabaena*^2^	98	EU110976	Florida Keys, mat on sediment	[38]
72-1	*Pseudanabaena*	98	EU196365	Florida Keys, NN dry rocks, mat on *Montastraea cavernosa*	[38]
63a-1	*Synechococcus*^2^	98	EU743972	Florida Keys, mat on sediment	[33]
63a-3	*Synechococcus*	98	EU743971	Florida Keys, mat on sediment	[33]

1 This strain, identified morphologically as *Phormidium* was subsequently shown using molecular techniques to belong to *Geitlerinema*;

2 These strains were not tested using hydrophilic extracts, see text;

3 Not sequenced;

4 Identified using classical taxonomic criteria because the BLAST search did not provide a species level identification.
